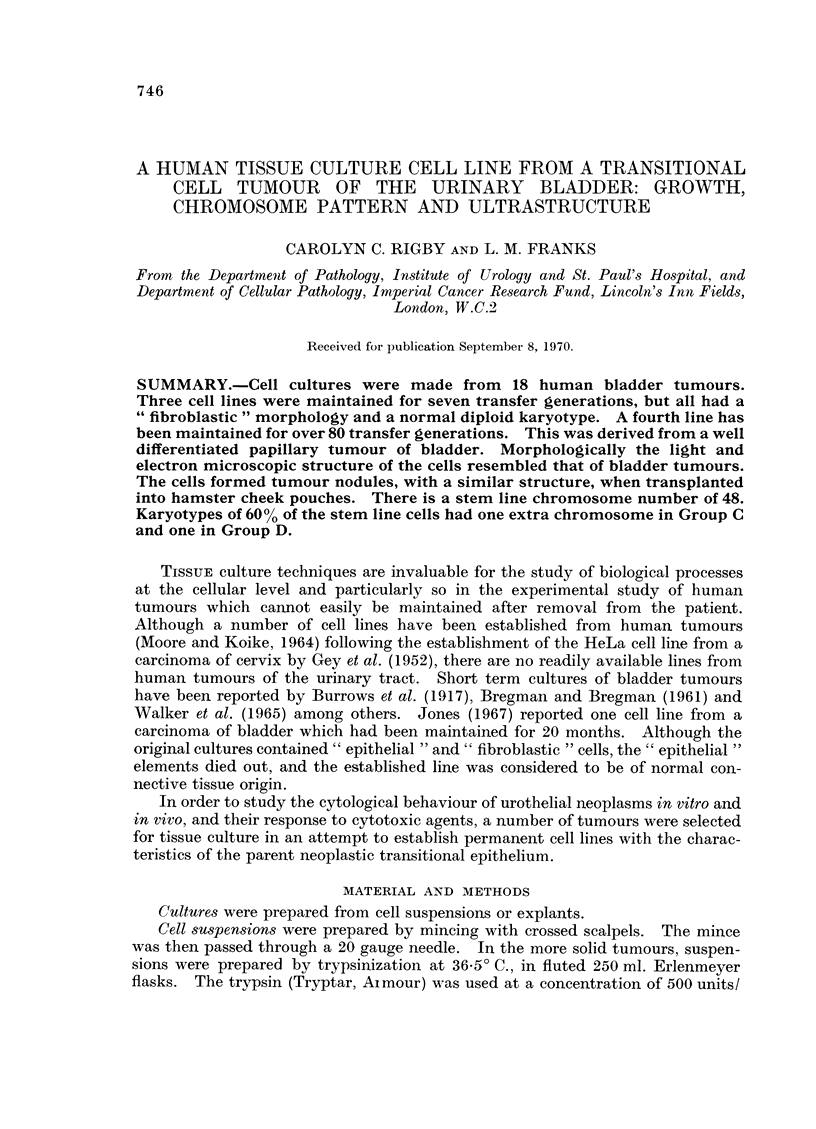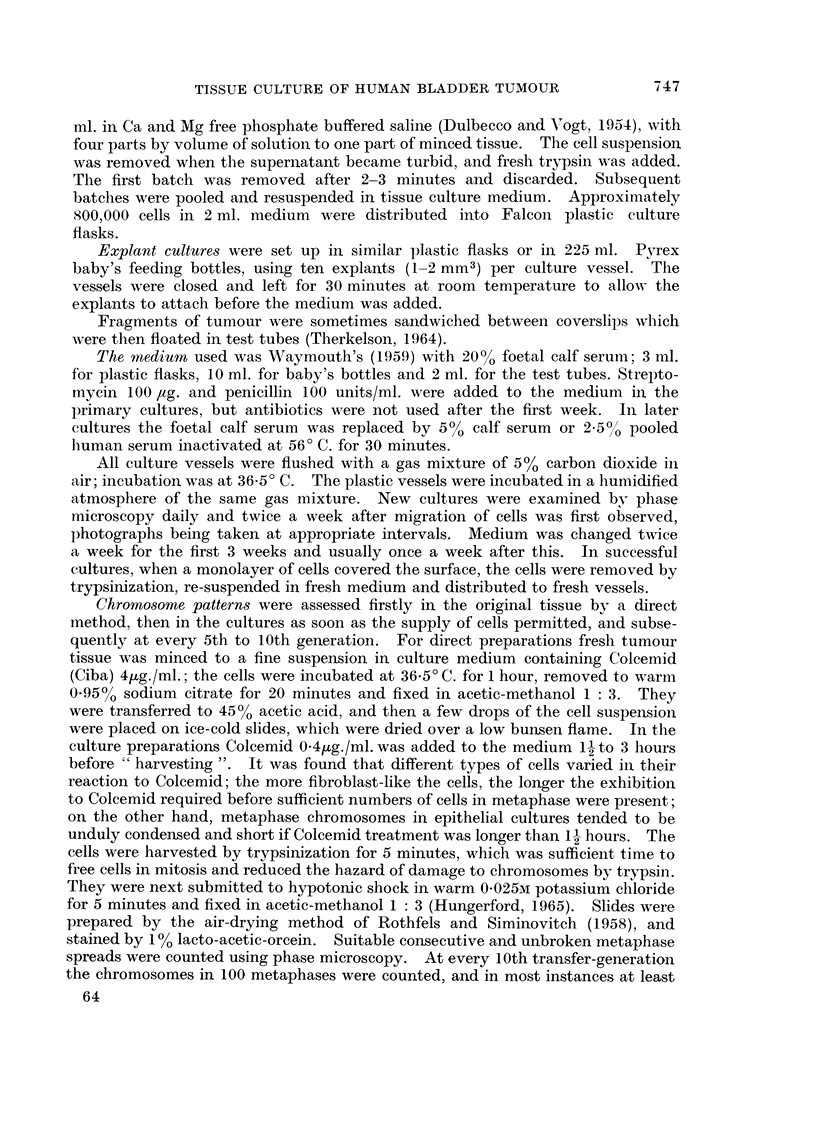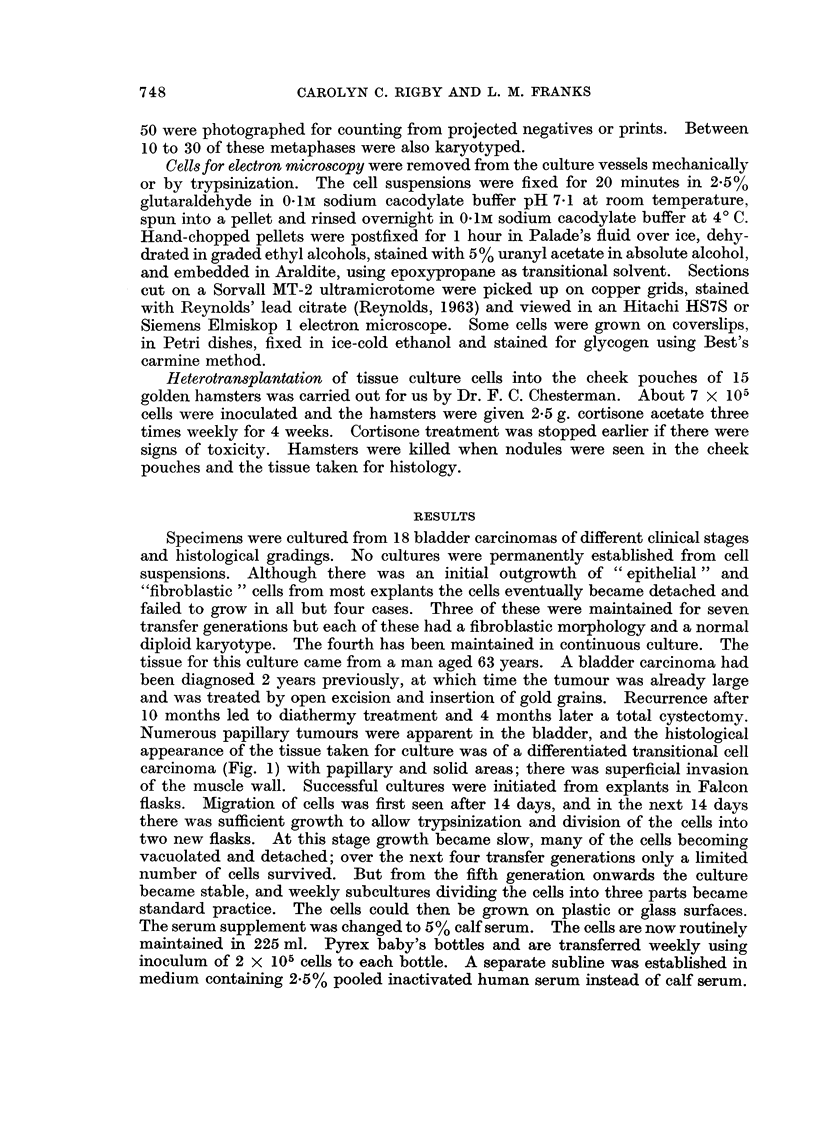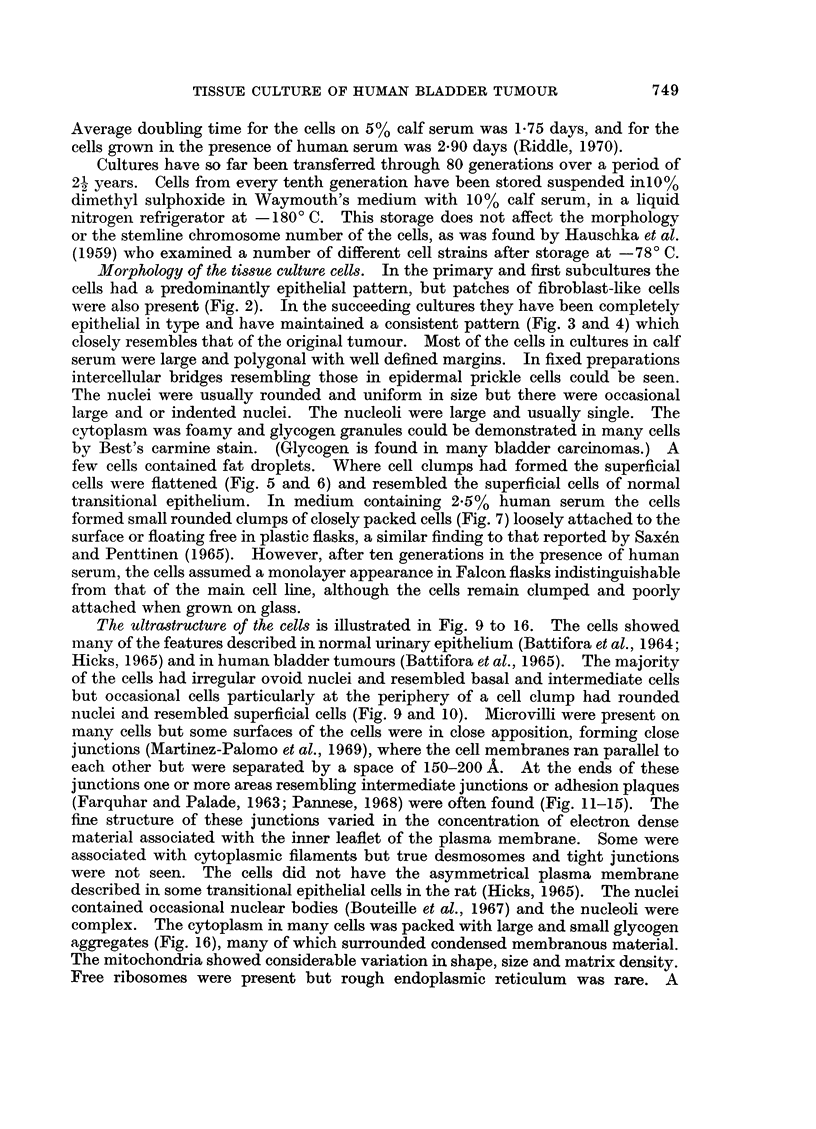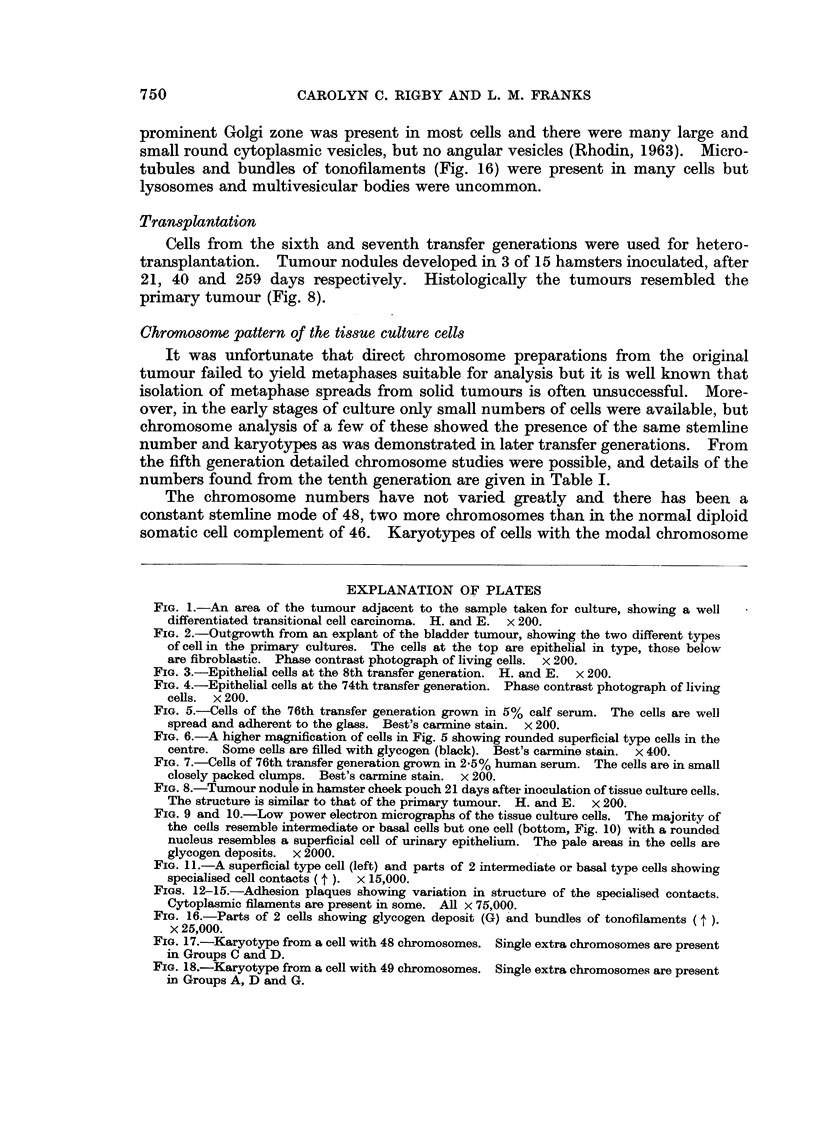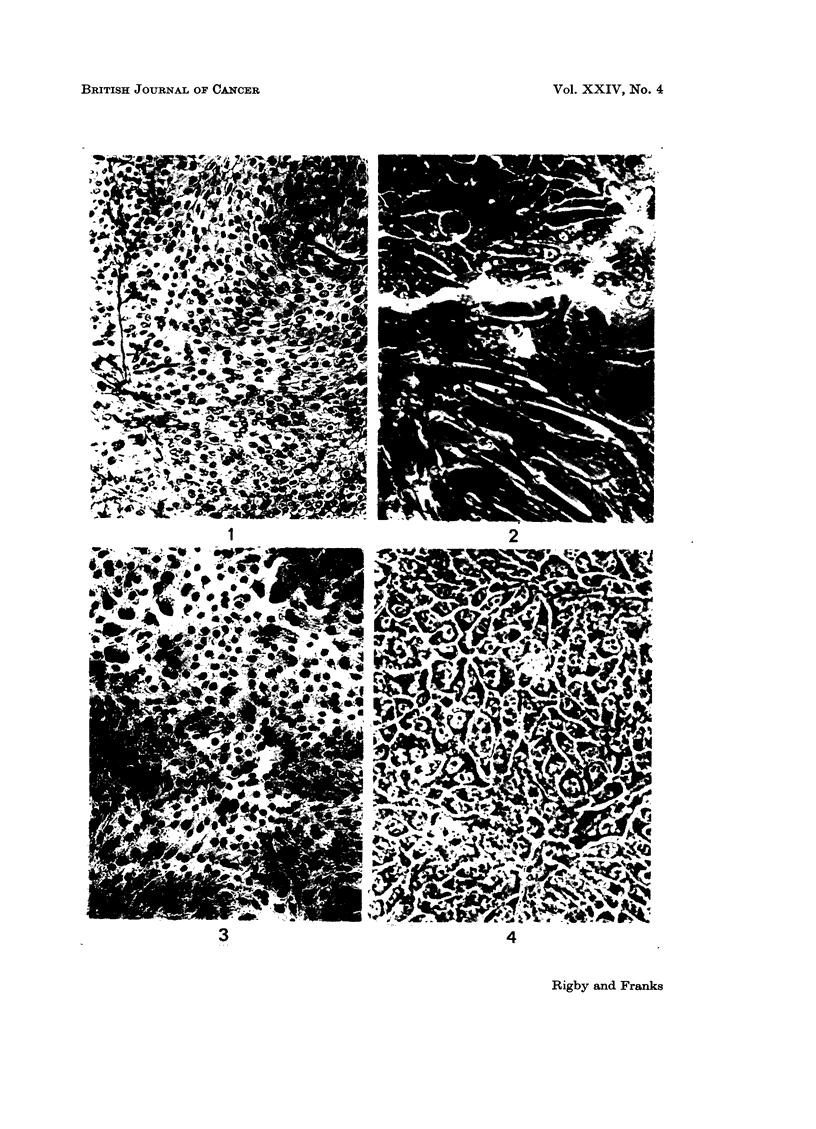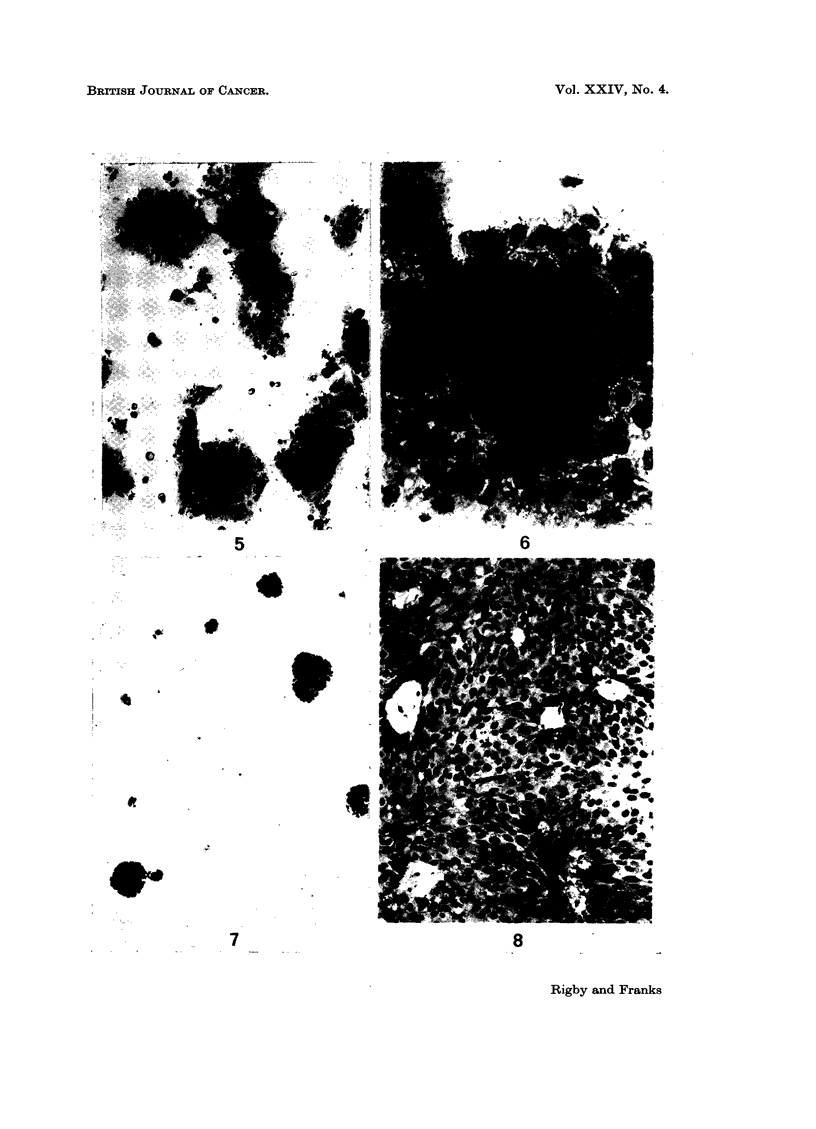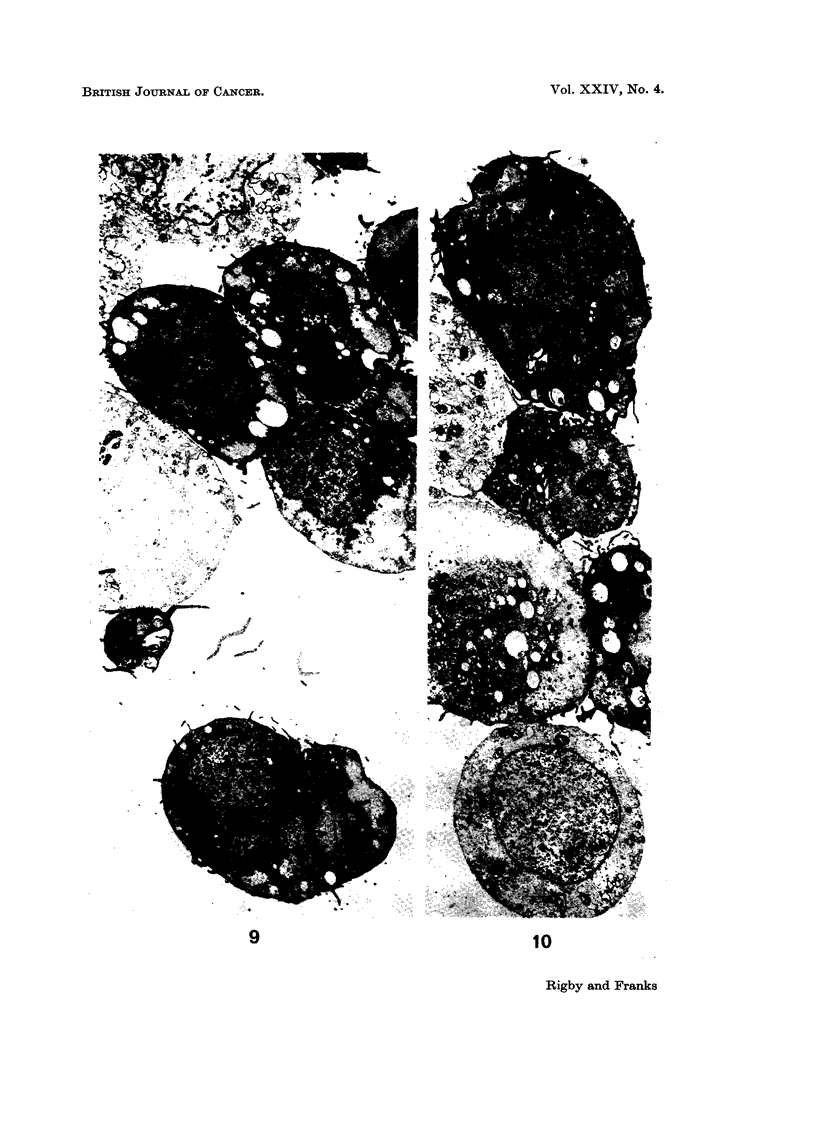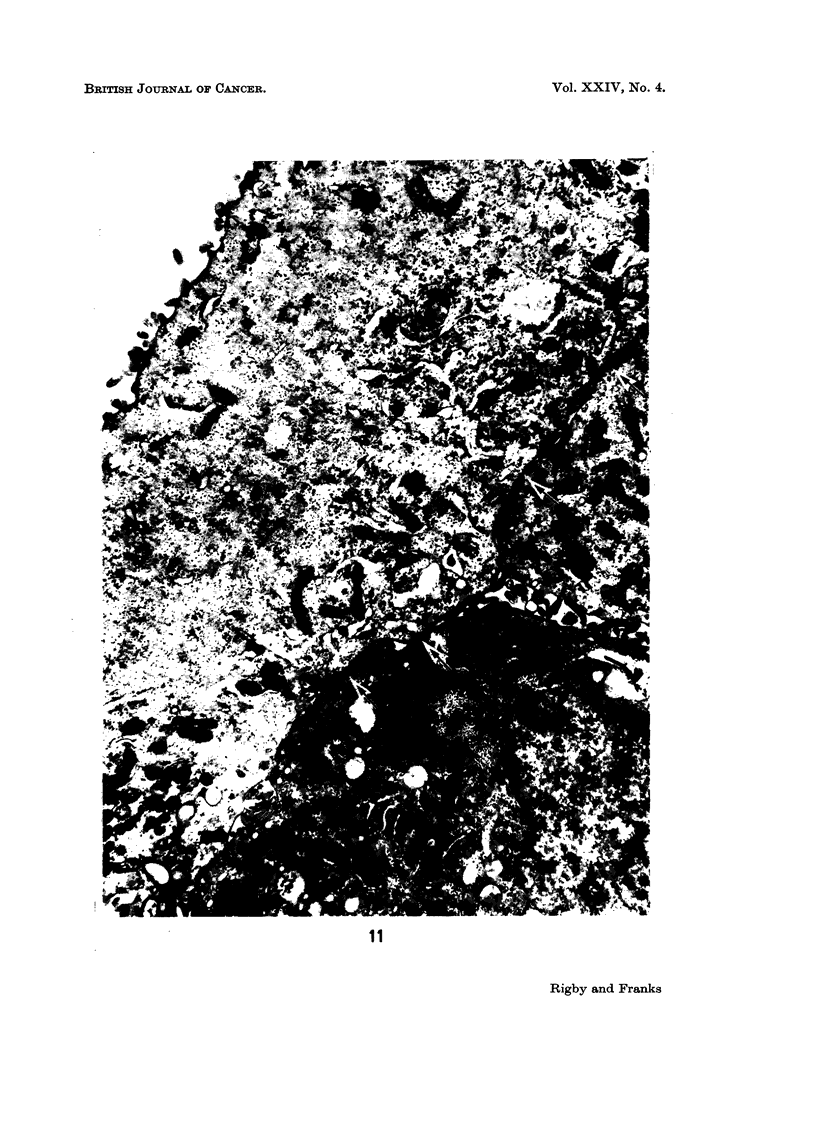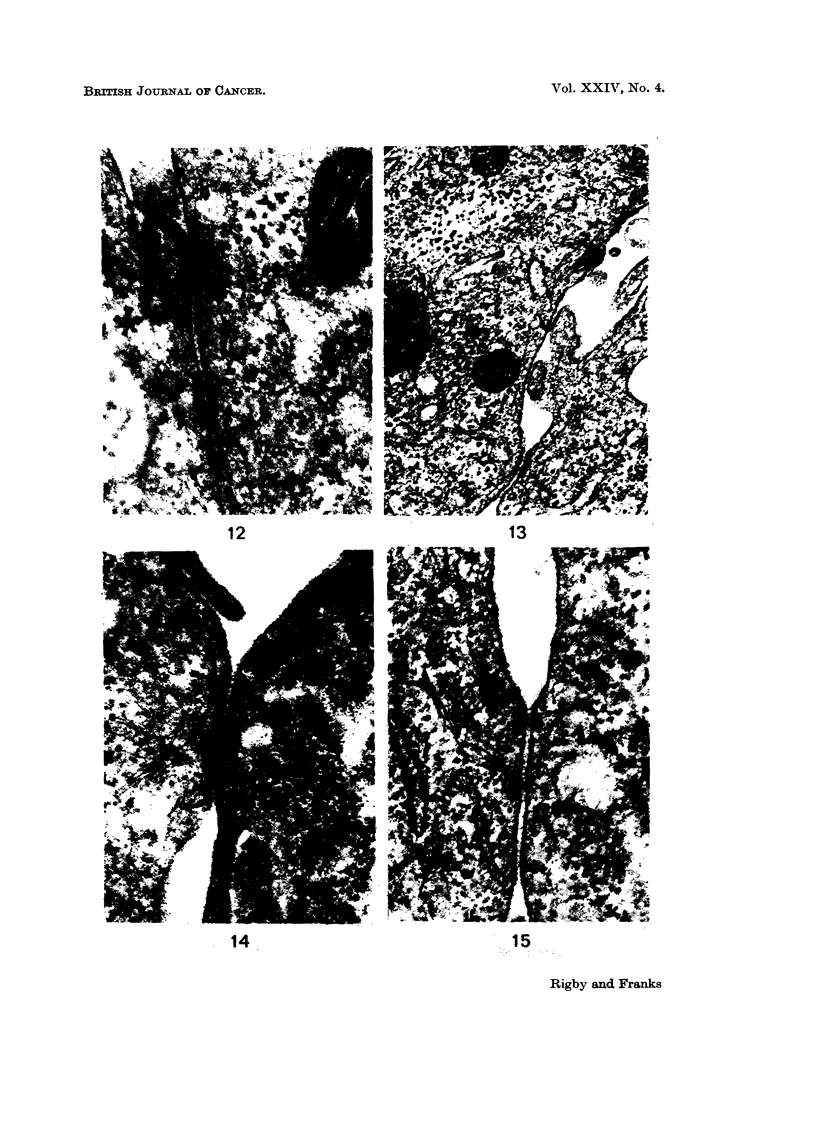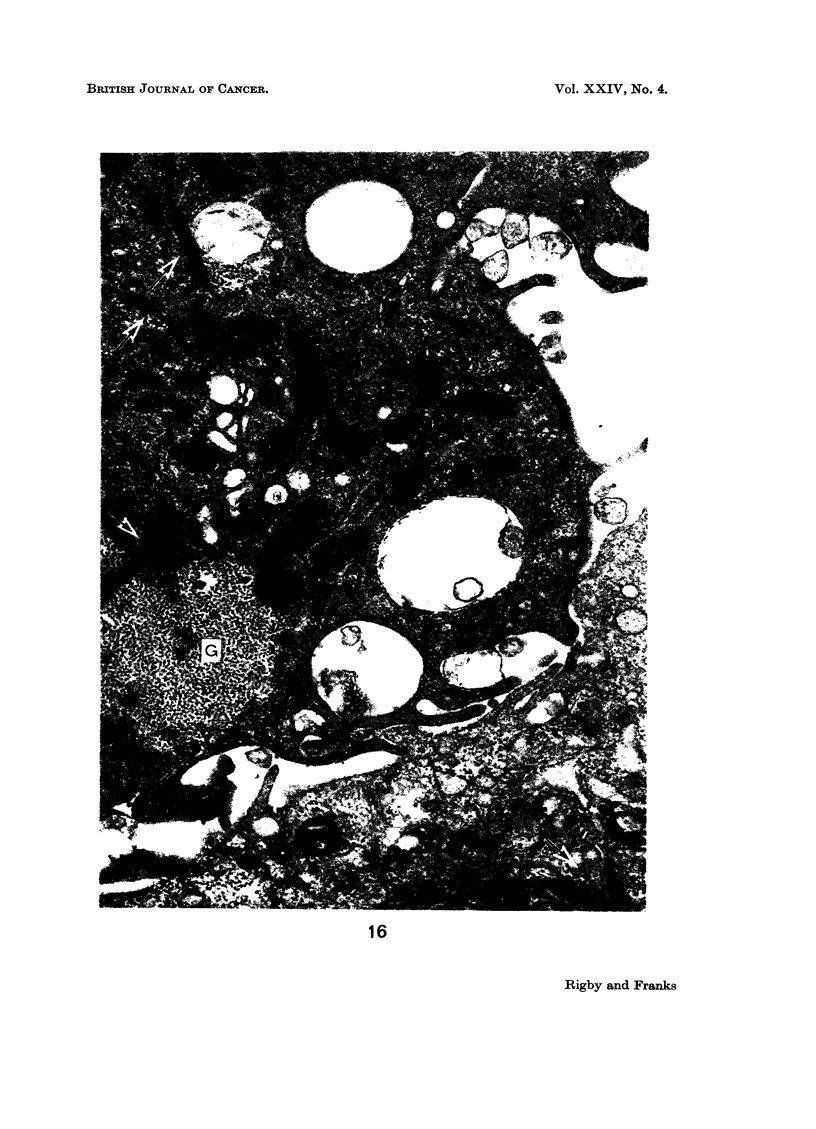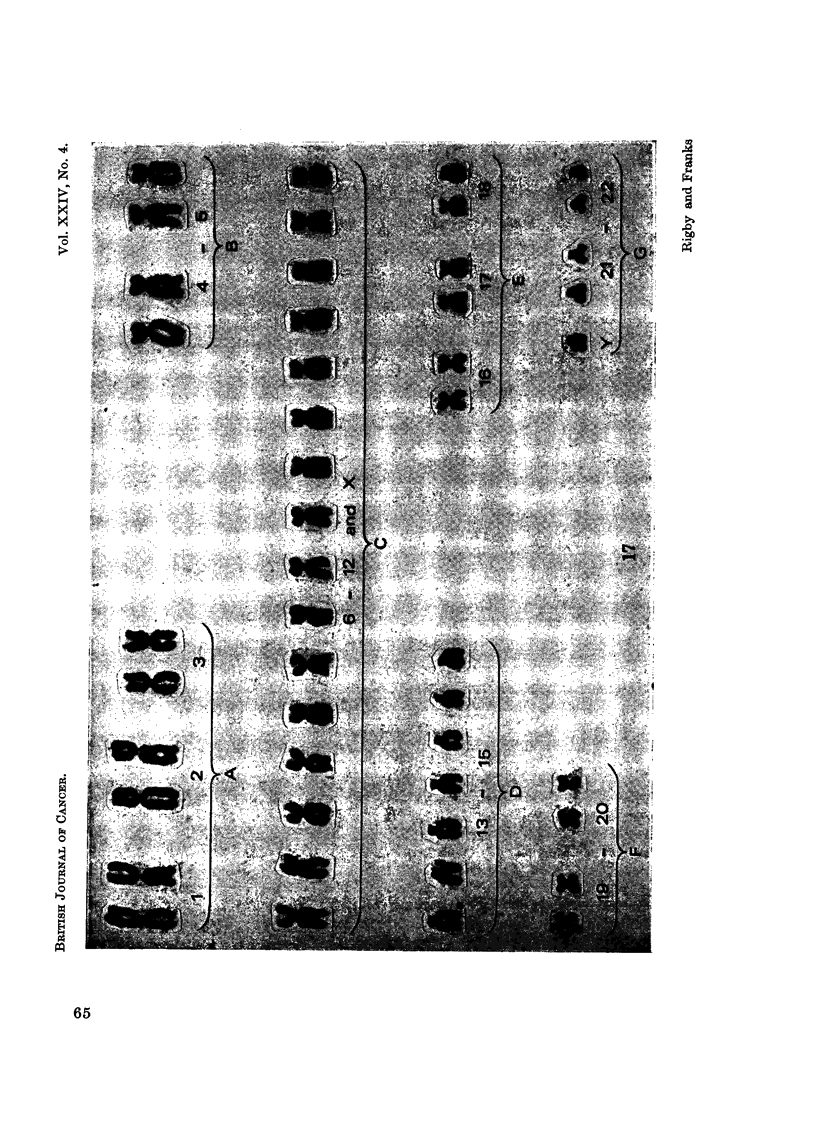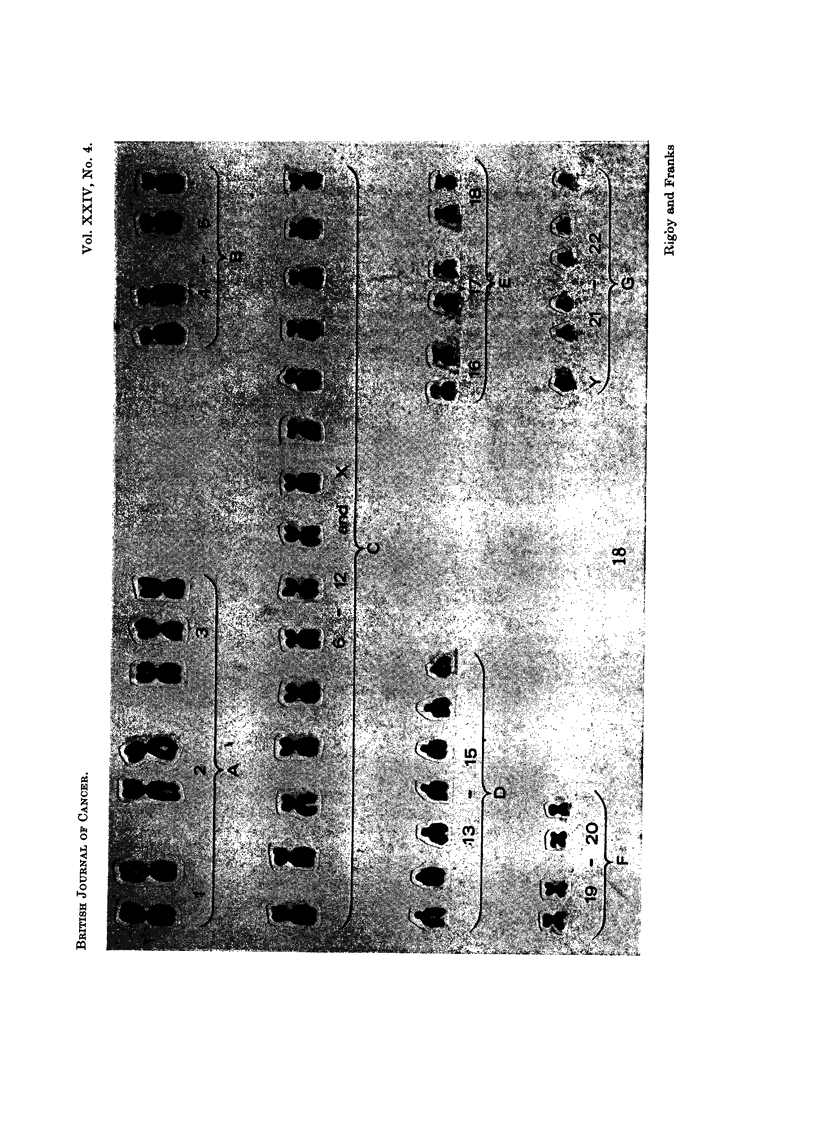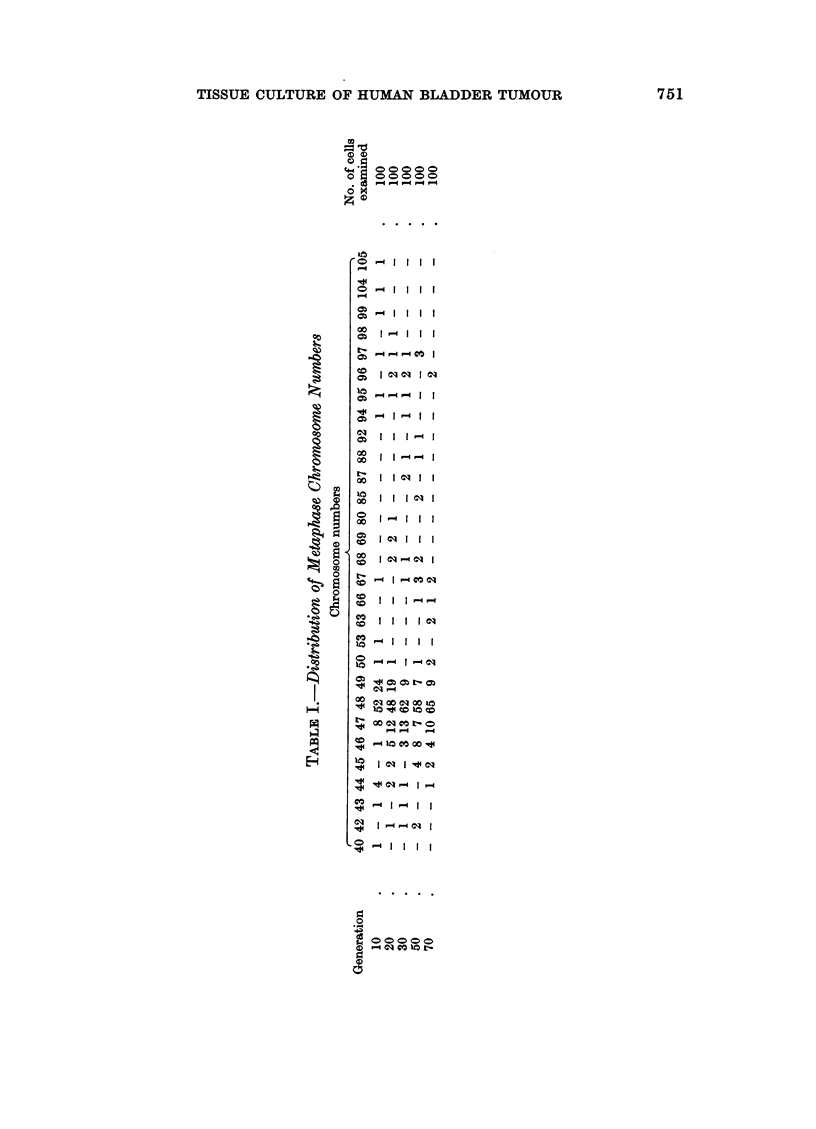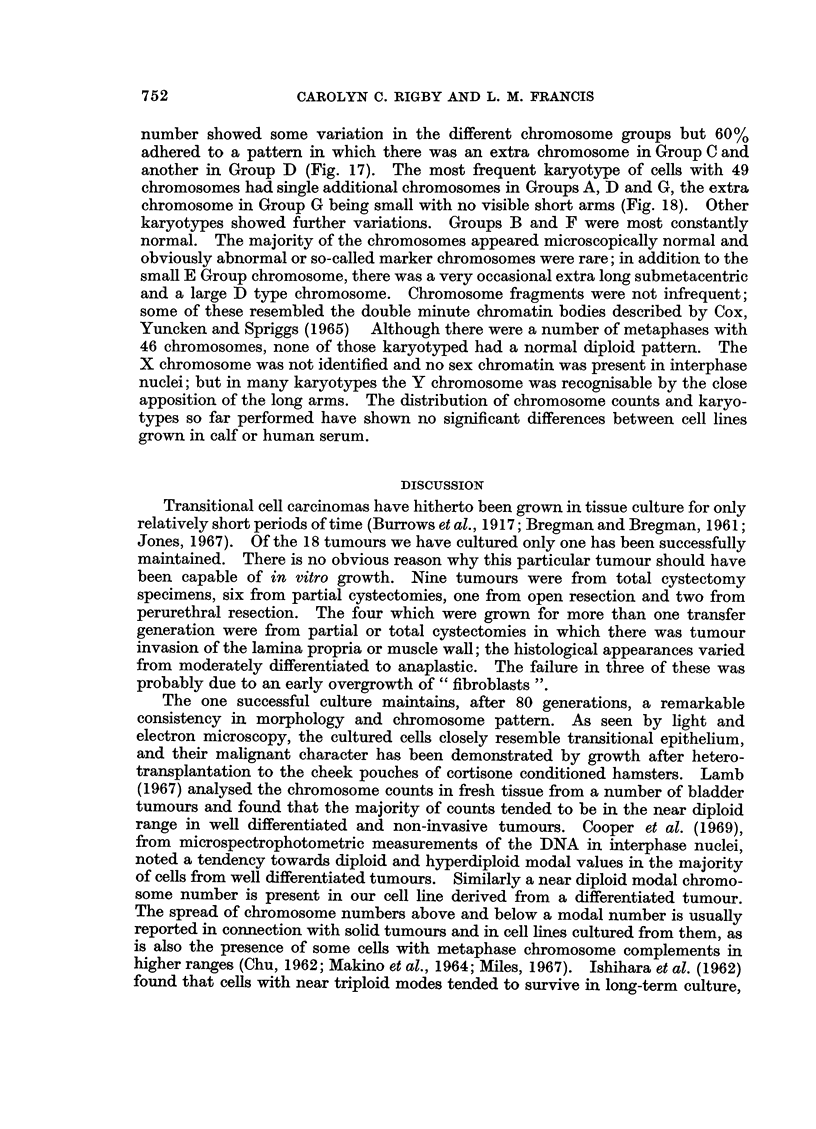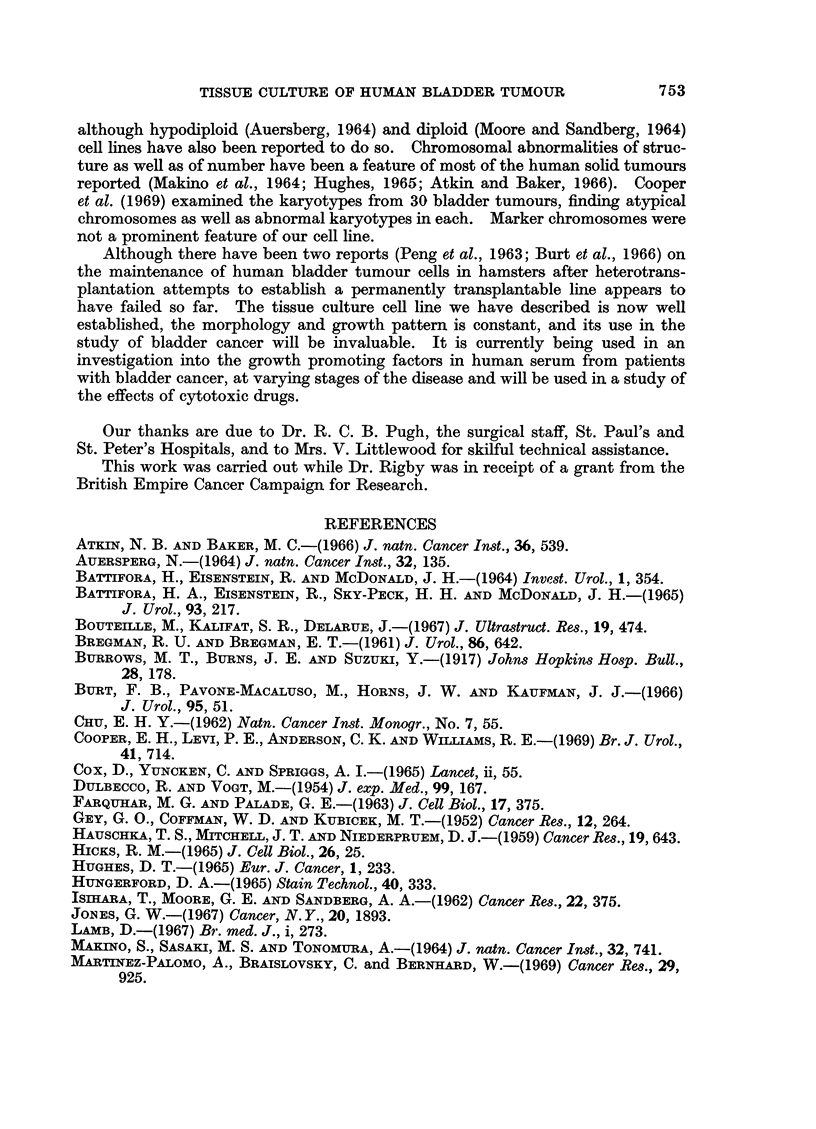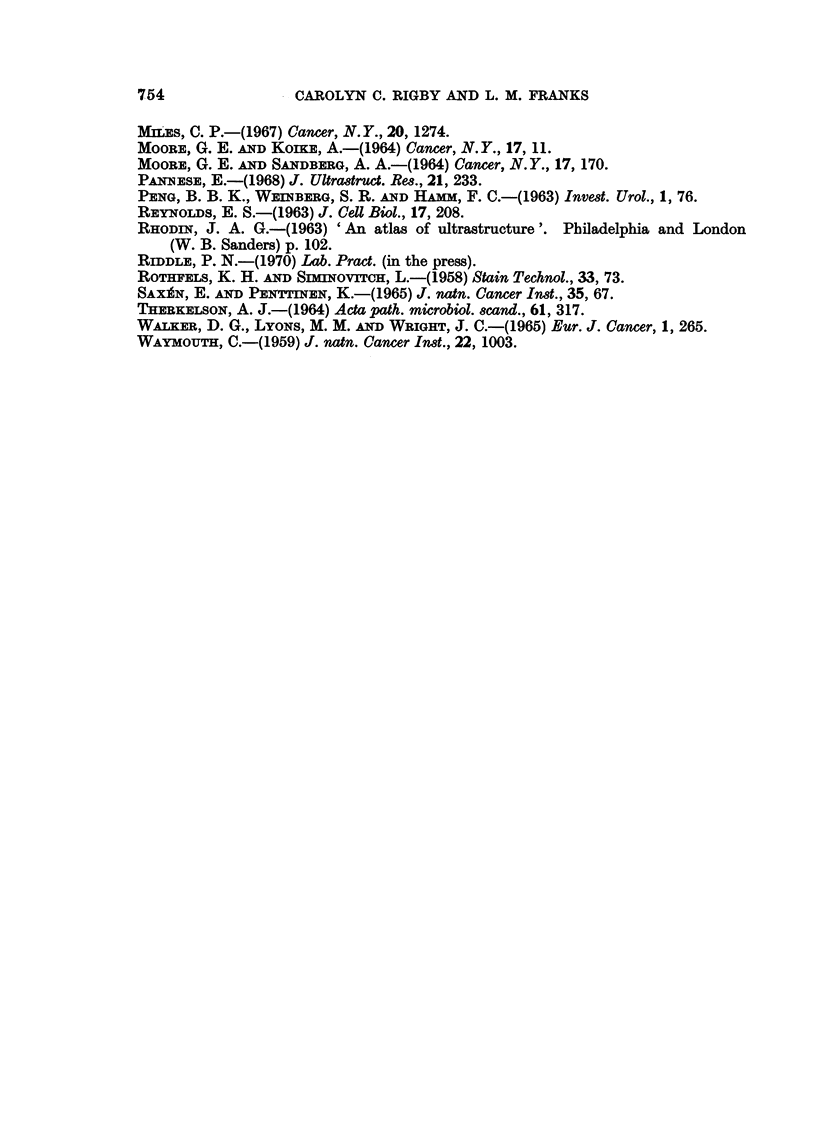# A Human Tissue Culture Cell Line from a Transitional Cell Tumour of the Urinary Bladder: Growth, Chromosome Pattern and Ultrastructure

**DOI:** 10.1038/bjc.1970.89

**Published:** 1970-12

**Authors:** Carolyn C. Rigby, L. M. Franks

## Abstract

**Images:**


### A HUMAN TISSUE CULTURE CELL LINE FROM A TRANSITIONAL CELL TUMOUR OF THE URINARY BLADDER: GROWTH, CHROMOSOME PATTERN AND ULTRASTRUCTURE

CAROLYN C. RIGBY AND L. M. FRANKS

*From the Department of Pathology, Institute of Urology and St. Paul's Hospital, and Department of Cellular Pathology, Imperial Cancer Research Fund, Lincoln's Inn Fields, London, W.C.2*

Received for publication September 8, 1970.

**SUMMARY.—Cell cultures were made from 18 human bladder tumours. Three cell lines were maintained for seven transfer generations, but all had a " fibroblastic " morphology and a normal diploid karyotype. A fourth line has been maintained for over 80 transfer generations. This was derived from a well differentiated papillary tumour of bladder. Morphologically the light and electron microscopic structure of the cells resembled that of bladder tumours. The cells formed tumour nodules, with a similar structure, when transplanted into hamster cheek pouches. There is a stem line chromosome number of 48. Karyotypes of 60% of the stem line cells had one extra chromosome in Group C and one in Group D.**

TISSUE culture techniques are invaluable for the study of biological processes at the cellular level and particularly so in the experimental study of human tumours which cannot easily be maintained after removal from the patient. Although a number of cell lines have been established from human tumours (Moore and Koike, 1964) following the establishment of the HeLa cell line from a carcinoma of cervix by Gey *et al.* (1952), there are no readily available lines from human tumours of the urinary tract. Short term cultures of bladder tumours have been reported by Burrows *et al.* (1917), Bregman and Bregman (1961) and Walker *et al.* (1965) among others. Jones (1967) reported one cell line from a carcinoma of bladder which had been maintained for 20 months. Although the original cultures contained " epithelial " and " fibroblastic " cells, the " epithelial " elements died out, and the established line was considered to be of normal connective tissue origin.

In order to study the cytological behaviour of urothelial neoplasms *in vitro* and *in vivo*, and their response to cytotoxic agents, a number of tumours were selected for tissue culture in an attempt to establish permanent cell lines with the characteristics of the parent neoplastic transitional epithelium.

### MATERIAL AND METHODS

*Cultures* were prepared from cell suspensions or explants.

*Cell suspensions* were prepared by mincing with crossed scalpels. The mince was then passed through a 20 gauge needle. In the more solid tumours, suspensions were prepared by trypsinization at 36·5° C., in fluted 250 ml. Erlenmeyer flasks. The trypsin (Tryptar, Aımour) was used at a concentration of 500 units/

ml. in Ca and Mg free phosphate buffered saline (Dulbecco and Vogt, 1954), with four parts by volume of solution to one part of minced tissue. The cell suspension was removed when the supernatant became turbid, and fresh trypsin was added. The first batch was removed after 2–3 minutes and discarded. Subsequent batches were pooled and resuspended in tissue culture medium. Approximately 800,000 cells in 2 ml. medium were distributed into Falcon plastic culture flasks.

*Explant cultures* were set up in similar plastic flasks or in 225 ml. Pyrex baby's feeding bottles, using ten explants (1–2 $mm^3$) per culture vessel. The vessels were closed and left for 30 minutes at room temperature to allow the explants to attach before the medium was added.

Fragments of tumour were sometimes sandwiched between coverslips which were then floated in test tubes (Therkelson, 1964).

*The medium* used was Waymouth's (1959) with 20% foetal calf serum; 3 ml. for plastic flasks, 10 ml. for baby's bottles and 2 ml. for the test tubes. Streptomycin 100 $\mu$g. and penicillin 100 units/ml. were added to the medium in the primary cultures, but antibiotics were not used after the first week. In later cultures the foetal calf serum was replaced by 5% calf serum or 2·5% pooled human serum inactivated at 56° C. for 30 minutes.

All culture vessels were flushed with a gas mixture of 5% carbon dioxide in air; incubation was at 36·5° C. The plastic vessels were incubated in a humidified atmosphere of the same gas mixture. New cultures were examined by phase microscopy daily and twice a week after migration of cells was first observed, photographs being taken at appropriate intervals. Medium was changed twice a week for the first 3 weeks and usually once a week after this. In successful cultures, when a monolayer of cells covered the surface, the cells were removed by trypsinization, re-suspended in fresh medium and distributed to fresh vessels.

*Chromosome patterns* were assessed firstly in the original tissue by a direct method, then in the cultures as soon as the supply of cells permitted, and subsequently at every 5th to 10th generation. For direct preparations fresh tumour tissue was minced to a fine suspension in culture medium containing Colcemid (Ciba) 4$\mu$g./ml.; the cells were incubated at 36·5° C. for 1 hour, removed to warm 0·95% sodium citrate for 20 minutes and fixed in acetic-methanol 1 : 3. They were transferred to 45% acetic acid, and then a few drops of the cell suspension were placed on ice-cold slides, which were dried over a low bunsen flame. In the culture preparations Colcemid 0·4$\mu$g./ml. was added to the medium $1\frac{1}{2}$ to 3 hours before " harvesting ". It was found that different types of cells varied in their reaction to Colcemid; the more fibroblast-like the cells, the longer the exhibition to Colcemid required before sufficient numbers of cells in metaphase were present; on the other hand, metaphase chromosomes in epithelial cultures tended to be unduly condensed and short if Colcemid treatment was longer than $1\frac{1}{2}$ hours. The cells were harvested by trypsinization for 5 minutes, which was sufficient time to free cells in mitosis and reduced the hazard of damage to chromosomes by trypsin. They were next submitted to hypotonic shock in warm 0·025M potassium chloride for 5 minutes and fixed in acetic-methanol 1 : 3 (Hungerford, 1965). Slides were prepared by the air-drying method of Rothfels and Siminovitch (1958), and stained by 1% lacto-acetic-orcein. Suitable consecutive and unbroken metaphase spreads were counted using phase microscopy. At every 10th transfer-generation the chromosomes in 100 metaphases were counted, and in most instances at least

50 were photographed for counting from projected negatives or prints. Between 10 to 30 of these metaphases were also karyotyped.

*Cells for electron microscopy* were removed from the culture vessels mechanically or by trypsinization. The cell suspensions were fixed for 20 minutes in 2·5% glutaraldehyde in 0·1M sodium cacodylate buffer pH 7·1 at room temperature, spun into a pellet and rinsed overnight in 0·1M sodium cacodylate buffer at 4° C. Hand-chopped pellets were postfixed for 1 hour in Palade's fluid over ice, dehydrated in graded ethyl alcohols, stained with 5% uranyl acetate in absolute alcohol, and embedded in Araldite, using epoxypropane as transitional solvent. Sections cut on a Sorvall MT-2 ultramicrotome were picked up on copper grids, stained with Reynolds' lead citrate (Reynolds, 1963) and viewed in an Hitachi HS7S or Siemens Elmiskop 1 electron microscope. Some cells were grown on coverslips, in Petri dishes, fixed in ice-cold ethanol and stained for glycogen using Best's carmine method.

*Heterotransplantation* of tissue culture cells into the cheek pouches of 15 golden hamsters was carried out for us by Dr. F. C. Chesterman. About $7 \times 10^5$ cells were inoculated and the hamsters were given 2·5 g. cortisone acetate three times weekly for 4 weeks. Cortisone treatment was stopped earlier if there were signs of toxicity. Hamsters were killed when nodules were seen in the cheek pouches and the tissue taken for histology.

### RESULTS

Specimens were cultured from 18 bladder carcinomas of different clinical stages and histological gradings. No cultures were permanently established from cell suspensions. Although there was an initial outgrowth of "epithelial" and "fibroblastic" cells from most explants the cells eventually became detached and failed to grow in all but four cases. Three of these were maintained for seven transfer generations but each of these had a fibroblastic morphology and a normal diploid karyotype. The fourth has been maintained in continuous culture. The tissue for this culture came from a man aged 63 years. A bladder carcinoma had been diagnosed 2 years previously, at which time the tumour was already large and was treated by open excision and insertion of gold grains. Recurrence after 10 months led to diathermy treatment and 4 months later a total cystectomy. Numerous papillary tumours were apparent in the bladder, and the histological appearance of the tissue taken for culture was of a differentiated transitional cell carcinoma (Fig. 1) with papillary and solid areas; there was superficial invasion of the muscle wall. Successful cultures were initiated from explants in Falcon flasks. Migration of cells was first seen after 14 days, and in the next 14 days there was sufficient growth to allow trypsinization and division of the cells into two new flasks. At this stage growth became slow, many of the cells becoming vacuolated and detached; over the next four transfer generations only a limited number of cells survived. But from the fifth generation onwards the culture became stable, and weekly subcultures dividing the cells into three parts became standard practice. The cells could then be grown on plastic or glass surfaces. The serum supplement was changed to 5% calf serum. The cells are now routinely maintained in 225 ml. Pyrex baby's bottles and are transferred weekly using inoculum of $2 \times 10^5$ cells to each bottle. A separate subline was established in medium containing 2·5% pooled inactivated human serum instead of calf serum.

Average doubling time for the cells on 5% calf serum was 1·75 days, and for the cells grown in the presence of human serum was 2·90 days (Riddle, 1970).

Cultures have so far been transferred through 80 generations over a period of 2½ years. Cells from every tenth generation have been stored suspended in10% dimethyl sulphoxide in Waymouth's medium with 10% calf serum, in a liquid nitrogen refrigerator at −180° C. This storage does not affect the morphology or the stemline chromosome number of the cells, as was found by Hauschka *et al.* (1959) who examined a number of different cell strains after storage at −78° C.

*Morphology of the tissue culture cells.* In the primary and first subcultures the cells had a predominantly epithelial pattern, but patches of fibroblast-like cells were also present (Fig. 2). In the succeeding cultures they have been completely epithelial in type and have maintained a consistent pattern (Fig. 3 and 4) which closely resembles that of the original tumour. Most of the cells in cultures in calf serum were large and polygonal with well defined margins. In fixed preparations intercellular bridges resembling those in epidermal prickle cells could be seen. The nuclei were usually rounded and uniform in size but there were occasional large and or indented nuclei. The nucleoli were large and usually single. The cytoplasm was foamy and glycogen granules could be demonstrated in many cells by Best's carmine stain. (Glycogen is found in many bladder carcinomas.) A few cells contained fat droplets. Where cell clumps had formed the superficial cells were flattened (Fig. 5 and 6) and resembled the superficial cells of normal transitional epithelium. In medium containing 2·5% human serum the cells formed small rounded clumps of closely packed cells (Fig. 7) loosely attached to the surface or floating free in plastic flasks, a similar finding to that reported by Saxén and Penttinen (1965). However, after ten generations in the presence of human serum, the cells assumed a monolayer appearance in Falcon flasks indistinguishable from that of the main cell line, although the cells remain clumped and poorly attached when grown on glass.

*The ultrastructure of the cells* is illustrated in Fig. 9 to 16. The cells showed many of the features described in normal urinary epithelium (Battifora *et al.*, 1964; Hicks, 1965) and in human bladder tumours (Battifora *et al.*, 1965). The majority of the cells had irregular ovoid nuclei and resembled basal and intermediate cells but occasional cells particularly at the periphery of a cell clump had rounded nuclei and resembled superficial cells (Fig. 9 and 10). Microvilli were present on many cells but some surfaces of the cells were in close apposition, forming close junctions (Martinez-Palomo *et al.*, 1969), where the cell membranes ran parallel to each other but were separated by a space of 150–200 Å. At the ends of these junctions one or more areas resembling intermediate junctions or adhesion plaques (Farquhar and Palade, 1963; Pannese, 1968) were often found (Fig. 11–15). The fine structure of these junctions varied in the concentration of electron dense material associated with the inner leaflet of the plasma membrane. Some were associated with cytoplasmic filaments but true desmosomes and tight junctions were not seen. The cells did not have the asymmetrical plasma membrane described in some transitional epithelial cells in the rat (Hicks, 1965). The nuclei contained occasional nuclear bodies (Bouteille *et al.*, 1967) and the nucleoli were complex. The cytoplasm in many cells was packed with large and small glycogen aggregates (Fig. 16), many of which surrounded condensed membranous material. The mitochondria showed considerable variation in shape, size and matrix density. Free ribosomes were present but rough endoplasmic reticulum was rare. A

prominent Golgi zone was present in most cells and there were many large and small round cytoplasmic vesicles, but no angular vesicles (Rhodin, 1963). Microtubules and bundles of tonofilaments (Fig. 16) were present in many cells but lysosomes and multivesicular bodies were uncommon.

*Transplantation*

Cells from the sixth and seventh transfer generations were used for heterotransplantation. Tumour nodules developed in 3 of 15 hamsters inoculated, after 21, 40 and 259 days respectively. Histologically the tumours resembled the primary tumour (Fig. 8).

*Chromosome pattern of the tissue culture cells*

It was unfortunate that direct chromosome preparations from the original tumour failed to yield metaphases suitable for analysis but it is well known that isolation of metaphase spreads from solid tumours is often unsuccessful. Moreover, in the early stages of culture only small numbers of cells were available, but chromosome analysis of a few of these showed the presence of the same stemline number and karyotypes as was demonstrated in later transfer generations. From the fifth generation detailed chromosome studies were possible, and details of the numbers found from the tenth generation are given in Table I.

The chromosome numbers have not varied greatly and there has been a constant stemline mode of 48, two more chromosomes than in the normal diploid somatic cell complement of 46. Karyotypes of cells with the modal chromosome

---

EXPLANATION OF PLATES

Fig. 1.—An area of the tumour adjacent to the sample taken for culture, showing a well differentiated transitional cell carcinoma. H. and E. × 200.

Fig. 2.—Outgrowth from an explant of the bladder tumour, showing the two different types of cell in the primary cultures. The cells at the top are epithelial in type, those below are fibroblastic. Phase contrast photograph of living cells. × 200.

Fig. 3.—Epithelial cells at the 8th transfer generation. H. and E. × 200.

Fig. 4.—Epithelial cells at the 74th transfer generation. Phase contrast photograph of living cells. × 200.

Fig. 5.—Cells of the 76th transfer generation grown in 5% calf serum. The cells are well spread and adherent to the glass. Best's carmine stain. × 200.

Fig. 6.—A higher magnification of cells in Fig. 5 showing rounded superficial type cells in the centre. Some cells are filled with glycogen (black). Best's carmine stain. × 400.

Fig. 7.—Cells of 76th transfer generation grown in 2·5% human serum. The cells are in small closely packed clumps. Best's carmine stain. × 200.

Fig. 8.—Tumour nodule in hamster cheek pouch 21 days after inoculation of tissue culture cells. The structure is similar to that of the primary tumour. H. and E. × 200.

Fig. 9 and 10.—Low power electron micrographs of the tissue culture cells. The majority of the cells resemble intermediate or basal cells but one cell (bottom, Fig. 10) with a rounded nucleus resembles a superficial cell of urinary epithelium. The pale areas in the cells are glycogen deposits. × 2000.

Fig. 11.—A superficial type cell (left) and parts of 2 intermediate or basal type cells showing specialised cell contacts ( ↑ ). × 15,000.

Figs. 12–15.—Adhesion plaques showing variation in structure of the specialised contacts. Cytoplasmic filaments are present in some. All × 75,000.

Fig. 16.—Parts of 2 cells showing glycogen deposit (G) and bundles of tonofilaments ( ↑ ). × 25,000.

Fig. 17.—Karyotype from a cell with 48 chromosomes. Single extra chromosomes are present in Groups C and D.

Fig. 18.—Karyotype from a cell with 49 chromosomes. Single extra chromosomes are present in Groups A, D and G.

1

2

3

4

5 6 7 8

Rigby and Franks

9 10

Rigby and Franks

11

Rigby and Franks

12 13 14 15

Rigby and Franks

16

Rigby and Franks

Rigby and Franks

18

Rigby and Franks

TABLE I.—*Distribution of Metaphase Chromosome Numbers*

| Generation | | Chromosome numbers 40 | 42 | 43 | 44 | 45 | 46 | 47 | 48 | 49 | 50 | 53 | 63 | 66 | 67 | 68 | 69 | 80 | 85 | 87 | 88 | 92 | 94 | 95 | 96 | 97 | 98 | 99 | 104 | 105 | | No. of cells examined |
|---|---|---|---|---|---|---|---|---|---|---|---|---|---|---|---|---|---|---|---|---|---|---|---|---|---|---|---|---|---|---|---|---|
| 10 | . | 1 | – | 1 | 4 | – | 1 | 8 | 52 | 24 | 1 | 1 | – | – | 1 | – | – | – | – | – | – | – | 1 | 1 | – | 1 | – | 1 | 1 | 1 | . | 100 |
| 20 | . | – | 1 | – | 2 | 2 | 5 | 12 | 48 | 19 | 1 | – | – | – | – | 2 | 2 | 1 | – | – | – | – | – | 1 | 2 | 1 | 1 | – | – | – | . | 100 |
| 30 | . | – | 1 | 1 | 1 | – | 3 | 13 | 62 | 9 | – | – | – | – | 1 | 1 | – | – | – | 2 | 1 | – | 1 | 1 | 2 | 1 | – | – | – | – | . | 100 |
| 50 | . | – | 2 | – | – | 4 | 8 | 7 | 58 | 7 | 1 | – | – | 1 | 3 | 2 | – | – | 2 | – | 1 | 1 | – | – | – | 3 | – | – | – | – | . | 100 |
| 70 | . | – | – | – | 1 | 2 | 4 | 10 | 65 | 9 | 2 | – | 2 | 1 | 2 | – | – | – | – | – | – | – | – | – | 2 | – | – | – | – | – | . | 100 |

number showed some variation in the different chromosome groups but 60% adhered to a pattern in which there was an extra chromosome in Group C and another in Group D (Fig. 17). The most frequent karyotype of cells with 49 chromosomes had single additional chromosomes in Groups A, D and G, the extra chromosome in Group G being small with no visible short arms (Fig. 18). Other karyotypes showed further variations. Groups B and F were most constantly normal. The majority of the chromosomes appeared microscopically normal and obviously abnormal or so-called marker chromosomes were rare; in addition to the small E Group chromosome, there was a very occasional extra long submetacentric and a large D type chromosome. Chromosome fragments were not infrequent; some of these resembled the double minute chromatin bodies described by Cox, Yuncken and Spriggs (1965) Although there were a number of metaphases with 46 chromosomes, none of those karyotyped had a normal diploid pattern. The X chromosome was not identified and no sex chromatin was present in interphase nuclei; but in many karyotypes the Y chromosome was recognisable by the close apposition of the long arms. The distribution of chromosome counts and karyotypes so far performed have shown no significant differences between cell lines grown in calf or human serum.

### DISCUSSION

Transitional cell carcinomas have hitherto been grown in tissue culture for only relatively short periods of time (Burrows *et al.*, 1917; Bregman and Bregman, 1961; Jones, 1967). Of the 18 tumours we have cultured only one has been successfully maintained. There is no obvious reason why this particular tumour should have been capable of *in vitro* growth. Nine tumours were from total cystectomy specimens, six from partial cystectomies, one from open resection and two from perurethral resection. The four which were grown for more than one transfer generation were from partial or total cystectomies in which there was tumour invasion of the lamina propria or muscle wall; the histological appearances varied from moderately differentiated to anaplastic. The failure in three of these was probably due to an early overgrowth of " fibroblasts ".

The one successful culture maintains, after 80 generations, a remarkable consistency in morphology and chromosome pattern. As seen by light and electron microscopy, the cultured cells closely resemble transitional epithelium, and their malignant character has been demonstrated by growth after heterotransplantation to the cheek pouches of cortisone conditioned hamsters. Lamb (1967) analysed the chromosome counts in fresh tissue from a number of bladder tumours and found that the majority of counts tended to be in the near diploid range in well differentiated and non-invasive tumours. Cooper *et al.* (1969), from microspectrophotometric measurements of the DNA in interphase nuclei, noted a tendency towards diploid and hyperdiploid modal values in the majority of cells from well differentiated tumours. Similarly a near diploid modal chromosome number is present in our cell line derived from a differentiated tumour. The spread of chromosome numbers above and below a modal number is usually reported in connection with solid tumours and in cell lines cultured from them, as is also the presence of some cells with metaphase chromosome complements in higher ranges (Chu, 1962; Makino *et al.*, 1964; Miles, 1967). Ishihara *et al.* (1962) found that cells with near triploid modes tended to survive in long-term culture, although hypodiploid (Auersberg, 1964) and diploid (Moore and Sandberg, 1964) cell lines have also been reported to do so. Chromosomal abnormalities of structure as well as of number have been a feature of most of the human solid tumours reported (Makino *et al.*, 1964; Hughes, 1965; Atkin and Baker, 1966). Cooper *et al.* (1969) examined the karyotypes from 30 bladder tumours, finding atypical chromosomes as well as abnormal karyotypes in each. Marker chromosomes were not a prominent feature of our cell line.

Although there have been two reports (Peng *et al.*, 1963; Burt *et al.*, 1966) on the maintenance of human bladder tumour cells in hamsters after heterotransplantation attempts to establish a permanently transplantable line appears to have failed so far. The tissue culture cell line we have described is now well established, the morphology and growth pattern is constant, and its use in the study of bladder cancer will be invaluable. It is currently being used in an investigation into the growth promoting factors in human serum from patients with bladder cancer, at varying stages of the disease and will be used in a study of the effects of cytotoxic drugs.

Our thanks are due to Dr. R. C. B. Pugh, the surgical staff, St. Paul's and St. Peter's Hospitals, and to Mrs. V. Littlewood for skilful technical assistance.

This work was carried out while Dr. Rigby was in receipt of a grant from the British Empire Cancer Campaign for Research.

### REFERENCES

ATKIN, N. B. AND BAKER, M. C.—(1966) *J. natn. Cancer Inst.*, **36**, 539.
AUERSPERG, N.—(1964) *J. natn. Cancer Inst.*, **32**, 135.
BATTIFORA, H., EISENSTEIN, R. AND MCDONALD, J. H.—(1964) *Invest. Urol.*, **1**, 354.
BATTIFORA, H. A., EISENSTEIN, R., SKY-PECK, H. H. AND MCDONALD, J. H.—(1965) *J. Urol.*, **93**, 217.
BOUTEILLE, M., KALIFAT, S. R., DELARUE, J.—(1967) *J. Ultrastruct. Res.*, **19**, 474.
BREGMAN, R. U. AND BREGMAN, E. T.—(1961) *J. Urol.*, **86**, 642.
BURROWS, M. T., BURNS, J. E. AND SUZUKI, Y.—(1917) *Johns Hopkins Hosp. Bull.*, **28**, 178.
BURT, F. B., PAVONE-MACALUSO, M., HORNS, J. W. AND KAUFMAN, J. J.—(1966) *J. Urol.*, **95**, 51.
CHU, E. H. Y.—(1962) *Natn. Cancer Inst. Monogr.*, No. 7, 55.
COOPER, E. H., LEVI, P. E., ANDERSON, C. K. AND WILLIAMS, R. E.—(1969) *Br. J. Urol.*, **41**, 714.
COX, D., YUNCKEN, C. AND SPRIGGS, A. I.—(1965) *Lancet*, ii, 55.
DULBECCO, R. AND VOGT, M.—(1954) *J. exp. Med.*, **99**, 167.
FARQUHAR, M. G. AND PALADE, G. E.—(1963) *J. Cell Biol.*, **17**, 375.
GEY, G. O., COFFMAN, W. D. AND KUBICEK, M. T.—(1952) *Cancer Res.*, **12**, 264.
HAUSCHKA, T. S., MITCHELL, J. T. AND NIEDERPRUEM, D. J.—(1959) *Cancer Res.*, **19**, 643.
HICKS, R. M.—(1965) *J. Cell Biol.*, **26**, 25.
HUGHES, D. T.—(1965) *Eur. J. Cancer*, **1**, 233.
HUNGERFORD, D. A.—(1965) *Stain Technol.*, **40**, 333.
ISIHARA, T., MOORE, G. E. AND SANDBERG, A. A.—(1962) *Cancer Res.*, **22**, 375.
JONES, G. W.—(1967) *Cancer, N.Y.*, **20**, 1893.
LAMB, D.—(1967) *Br. med. J.*, i, 273.
MAKINO, S., SASAKI, M. S. AND TONOMURA, A.—(1964) *J. natn. Cancer Inst.*, **32**, 741.
MARTINEZ-PALOMO, A., BRAISLOVSKY, C. and BERNHARD, W.—(1969) *Cancer Res.*, **29**, 925.

MILES, C. P.—(1967) *Cancer, N.Y.*, **20**, 1274.
MOORE, G. E. AND KOIKE, A.—(1964) *Cancer, N.Y.*, **17**, 11.
MOORE, G. E. AND SANDBERG, A. A.—(1964) *Cancer, N.Y.*, **17**, 170.
PANNESE, E.—(1968) *J. Ultrastruct. Res.*, **21**, 233.
PENG, B. B. K., WEINBERG, S. R. AND HAMM, F. C.—(1963) *Invest. Urol.*, **1**, 76.
REYNOLDS, E. S.—(1963) *J. Cell Biol.*, **17**, 208.
RHODIN, J. A. G.—(1963) 'An atlas of ultrastructure'. Philadelphia and London (W. B. Sanders) p. 102.
RIDDLE, P. N.—(1970) *Lab. Pract.* (in the press).
ROTHFELS, K. H. AND SIMINOVITCH, L.—(1958) *Stain Technol.*, **33**, 73.
SAXÉN, E. AND PENTTINEN, K.—(1965) *J. natn. Cancer Inst.*, **35**, 67.
THERKELSON, A. J.—(1964) *Acta path. microbiol. scand.*, **61**, 317.
WALKER, D. G., LYONS, M. M. AND WRIGHT, J. C.—(1965) *Eur. J. Cancer*, **1**, 265.
WAYMOUTH, C.—(1959) *J. natn. Cancer Inst.*, **22**, 1003.